# Does fraud victimization affect a person’s long-term blood pressure?

**Published:** 2022-09

1.

**AUGUST 1, 2022** - New research published in the Journal of the American Geriatrics Society suggests that experiencing financial exploitation, fraudulent schemes, and scams may raise a person’s blood pressure. The study found that fraud victimization was linked with elevated blood pressure in men, but not in women.

The study included 1,200 older adults from the Rush Memory and Aging Project. During up to 11 years of annual observations, participants were asked about fraud victimization and underwent serial blood pressure measurements.

Men showed post-fraud elevations in blood pressure that, compounded over time, may portend poor health.

“These findings show that fraud victimization has important public health consequences and underscore the need for efforts to prevent exploitation,” said lead author Melissa Lamar, PhD, of Rush University Medical Center.

***Link to Study: https://onlinelibrary.wiley.com/doi/10.1111/jgs.17951***

***Full citation:** “Self-reported fraud victimization and objectively measured blood pressure: Sex differences in post-fraud cardiovascular health”Melissa Lamar, Lei Yu, Sue Leurgans PhD,Neelum T. Aggarwal MD,Robert S. Wilson PhD,S. Duke Han, David A. Bennett, Patricia Boyle. J Am Geriatr Soc.; Published Online: 03 August 2022 (DOI: 110.1111/jgs.17951).*

*Copyright © 2019 The Cochrane Collaboration. Published by John Wiley & Sons, Ltd., reproduced with permission.*

